# Real-Time Imaging of Mitochondrial ATP Dynamics Reveals the Metabolic Setting of Single Cells

**DOI:** 10.1016/j.celrep.2018.09.027

**Published:** 2018-10-09

**Authors:** Maria R. Depaoli, Felix Karsten, Corina T. Madreiter-Sokolowski, Christiane Klec, Benjamin Gottschalk, Helmut Bischof, Emrah Eroglu, Markus Waldeck-Weiermair, Thomas Simmen, Wolfgang F. Graier, Roland Malli

**Affiliations:** 1 Molecular Biology and Biochemistry, Gottfried Schatz Research Center, Medical University of Graz, Neue Stiftingtalstraße 6/6, 8010 Graz, Austria; 2 Division of Oncology, Research Unit for Long Non-coding RNAs and Genome Editing in Cancer, Medical University of Graz, Stiftingtalstraße 24, 8010 Graz, Austria; 3 BioTechMed Graz, Mozartgasse 12/II, 8010 Graz, Austria; 4 Department of Cell Biology, University of Alberta, Edmonton, AB T6G 2H7, Canada

## Abstract

Reprogramming of metabolic pathways determines cell functions and fate. In our work, we have used organelle-targeted ATP biosensors to evaluate cellular metabolic settings with high resolution in real time. Our data indicate that mitochondria dynamically supply ATP for glucose phosphorylation in a variety of cancer cell types. This hexokinase-dependent process seems to be reversed upon the removal of glucose or other hexose sugars. Our data further verify that mitochondria in cancer cells have increased ATP consumption. Similar subcellular ATP fluxes occurred in young mouse embryonic fibroblasts (MEFs). However, pancreatic beta cells, senescent MEFs, and MEFs lacking mitofusin 2 displayed completely different mitochondrial ATP dynamics, indicative of increased oxidative phosphorylation. Our findings add perspective to the variability of the cellular bioenergetics and demonstrate that live cell imaging of mitochondrial ATP dynamics is a powerful tool to evaluate metabolic flexibility and heterogeneity at a single-cell level.

## Introduction

Multiple cellular pathways converge to regulate the complex energy metabolism, which is a determinant for cell functions and fate (DeBerardinis and Thompson, 2012). As the nutrient availability varies, cells need to handle both abundance and lack of metabolizable substrates by reprogramming metabolic pathways (DeBerardinis and Chandel, 2016; Vander Heiden and DeBerardinis, 2017). A growing number of findings highlight that such processes are vital for cells to fulfill specific and essential functions (Gao et al., 2014; Goodpaster and Sparks, 2017; Ryall, 2013; Sousa et al., 2015). Cells of the immune system, for example, shift between different metabolic pathways in order to activate either inflammatory or anti-inflammatory responses (Van den Bossche et al., 2017). Metabolic reprogramming can also cause severe pathologies, such as inflammation (Kelly and O’Neill, 2015), neurodegeneration (Engel, 2016), and heart failure (Sun and Wang, 2016). Moreover, metabolic changes have been associated with tumorigenesis and cancer progression (Gentric et al., 2017; Vander Heiden and DeBerardinis, 2017). The energy metabolism of cancer cells is optimized to promote cell growth and proliferation and thereby distinguishes itself from most differentiated cells. Over the past decades, the metabolic reprogramming in cancer has been studied extensively (Gobbe and Herchuelz, 1989). Strikingly, it has been suggested that cancer might represent a metabolic disease, rather than a genetic one (Seyfried et al., 2014), emphasizing that metabolic alterations could be causative for tumor formation, a view that contrasts with the common opinion that DNA mutations initiate tumorigenesis (Haber and Fearon, 1998).

A common feature of many cancer cells and other rapidly proliferating cells (Brand and Hermfisse, 1997) is an increased uptake of glucose, which is subsequently fermented to lactate even in the presence of enough oxygen and fully functional mitochondria. This phenomenon, known as the “Warburg Effect” (Liberti and Locasale, 2016), was discovered more than 90 years ago, and its causes and consequences are still extensively investigated. Although conversion of glucose to lactate yields considerably less energy in the form of ATP per input glucose molecules compared to full glucose oxidation via mitochondrial respiration, cancer cells might benefit from low rates of oxidative phosphorylation (Vander Heiden et al., 2009). Oxygen consumption by mitochondria, coupled with electron transfer by the complexes of the respiratory chain, is often accompanied by the generation of reactive oxygen species (ROS) (Murphy, 2009), which have critical signaling functions (D’Autréaux and Toledano, 2007) but can also lead to cell damage and death (Panieri and Santoro, 2016). Hence, an important feature of cancer cell metabolism might be a fast and constant generation of high amounts of ATP, while maintaining a vital balance of ROS formation and signaling (Ogrunc, 2014). This implies that cancer cells must be metabolically flexible and able to switch between substrate sources in order to fill metabolite pools and optimize ATP generation and consumption (Porporato et al., 2018). However, our understanding of the dynamics of such processes on the level of single cells as well as the molecular mechanisms behind them is quite limited.

Recently, genetically encoded fluorescent probes for real-time imaging of specific cellular metabolites have been developed (e.g., Bilan et al., 2014; San Martín et al., 2014; Takanaga et al., 2008). Among these tools are Förster resonance energy transfer (FRET)-based ATP probes, referred to as “ATeams” (Imamura et al., 2009; Vishnu et al., 2014; Yoshida et al., 2017). ATeams are approved tools that enable visualizing spatiotemporal dynamics of intracellular ATP fluctuations and, thus, give insight into the metabolic activities of individual cells. Here, we used these fluorescent probes targeted to distinct cellular compartments in order to investigate the dynamics of intracellular ATP pools in response to acute glucose removal, glucose substitution, as well as mitochondrial toxins. With our imaging approach, we show that mitochondrial ATP is particularly subject to fluctuations following such interventions. Moreover, we introduce a meaningful imaging approach to investigate the metabolic activity and flexibility at the single-cell level, allowing us to characterize cancer cell metabolism, as well as to detect metabolic adaptations in response to cellular aging or gene knockout.

## Results

### Acute Glucose Starvation Causes Strong ATP Alterations within the Mitochondria of HeLa Cells

**Figure 1:**
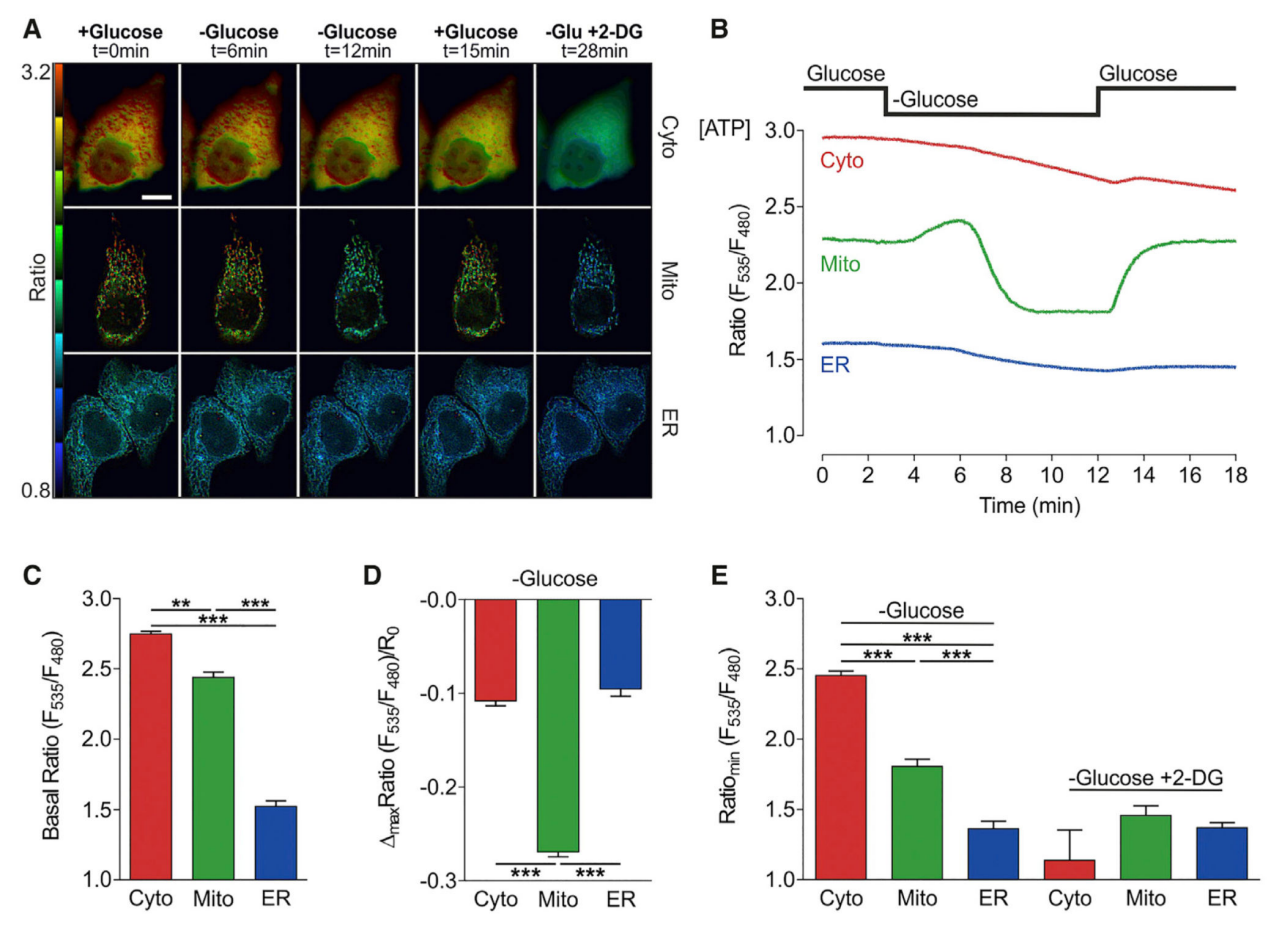
Acute Glucose Starvation Causes Strong ATP Alterations within Mitochondria of HeLa Cells (A) Representative FRET ratio images of cytosolic, mitochondrial, and ER-targeted ATP probes (ATeams) under basal conditions and at different time points after glucose depletion and subsequent 2-DG treatment. Scale bar represents 20 μm. See also Figures S1D and S1E and Video S1. (B) Representative single-cell responses to glucose deprivation of FRET ratio signals of cells expressing cytosolic (red), mitochondrial (green), and ER-targeted (blue) ATeams. (C) Basal FRET ratio values (mean, SEM) in the cytosol (red column, n = 5/42 cells), mitochondria (green column, n = 24/124 cells), and ER (blue column, n = 7/27 cells). **p < 0.01; ***p < 0.001. (D) Maximal changes (mean, SEM) of normalized FRET ratio signals in response to glucose removal (10 to 0 mM). ***p < 0.001. (E) Minimal FRET ratio values after glucose depletion (left columns) and upon addition of 10 mM 2-DG in the absence of glucose (right columns) in cells expressing cytosolic (red columns, n = 3/25 cells), mitochondria (green columns, n = 7/31 cells), or ER-targeted (blue columns, n = 7/31 cells) ATP probes. Mean, SEM. ***p < 0.001. See also Figures S1A–S1C.

To uncover the metabolic settings and flexibility of single cells, we utilized the rather simple protocol of glucose deprivation and its impact on organelle ATP levels. We started with HeLa cells, a frequently used standard cancer cell line (Masters, 2002), to investigate how dynamic glucose withdrawal might affect the intracellular ATP content of cancer cells. Cells were transfected with FRET-based ATP sensors targeted to the cytosol (Cyto, AT1.03) (Imamura et al., 2009), mitochondria (Mito, mtAT1.03) (Imamura et al., 2009), or endoplasmic reticulum (ER, ERAT1.03) (Vishnu et al., 2014), respectively (Figure 1; Video S1). Since all ATP sensors used in this study are ratiometric, FRET ratios were found to be independent of the expression levels of the probe (Figures S1A–S1C) and thus allow the assessment of intracellular ATP levels and changes. FRET ratio signals corresponding to intracellular ATP levels were measured over time in single cells exhibiting the correct localization of the sensors (Figure 1A). We observed that under basal conditions—in the presence of glucose—ATP levels remained constant and were the highest in the cytosol, slightly lower in the matrix of mitochondria, and the lowest in the ER lumen (Figures 1A–1C). Interestingly, the FRET ratio values of the mitochondria-targeted ATP sensor showed clear variations (Figures 1A and S1D) compared to the cytosolic and ER-targeted probes (Figures 1A and S1E), pointing to areas of high and low ATP levels within mitochondria. However, in this study we did not focus on local submitochondrial ATP gradients, but were interested in global ATP dynamics within the various cellular compartments upon glucose depletion (Figures 1A, 1B, 1D, and 1E). In mitochondria, glucose removal induced a transient rise of global matrix ATP followed by a fast and prominent decline within minutes (Figures 1A and 1B). Mitochondrial ATP levels quickly recovered upon glucose readdition to HeLa cells (Figures 1A and 1B). On the other hand, the high cytosolic and low ER ATP levels changed only moderately and rather slowly when glucose was removed or readded (Figures 1A and 1B). Cell treatment with 2-deoxyglucose (2-DG), a glucose analog that inhibits glycolysis following ATP dependent phosphorylation (Xi et al., 2014), reduced ATP levels within mitochondria more strongly than glucose withdrawal alone, and also markedly reduced cytosolic ATP levels (Figures 1A and 1E). These experiments showed that glucose deprivation mostly affects mitochondrial ATP levels, resulting in an initial increase and a subsequent depletion of ATP within this organelle.

### Transient Mitochondrial ATP Increase upon Glucose Deprivation Depends on Mitochondria-Located Hexokinase 1 and 2 Activities

**Figure 2:**
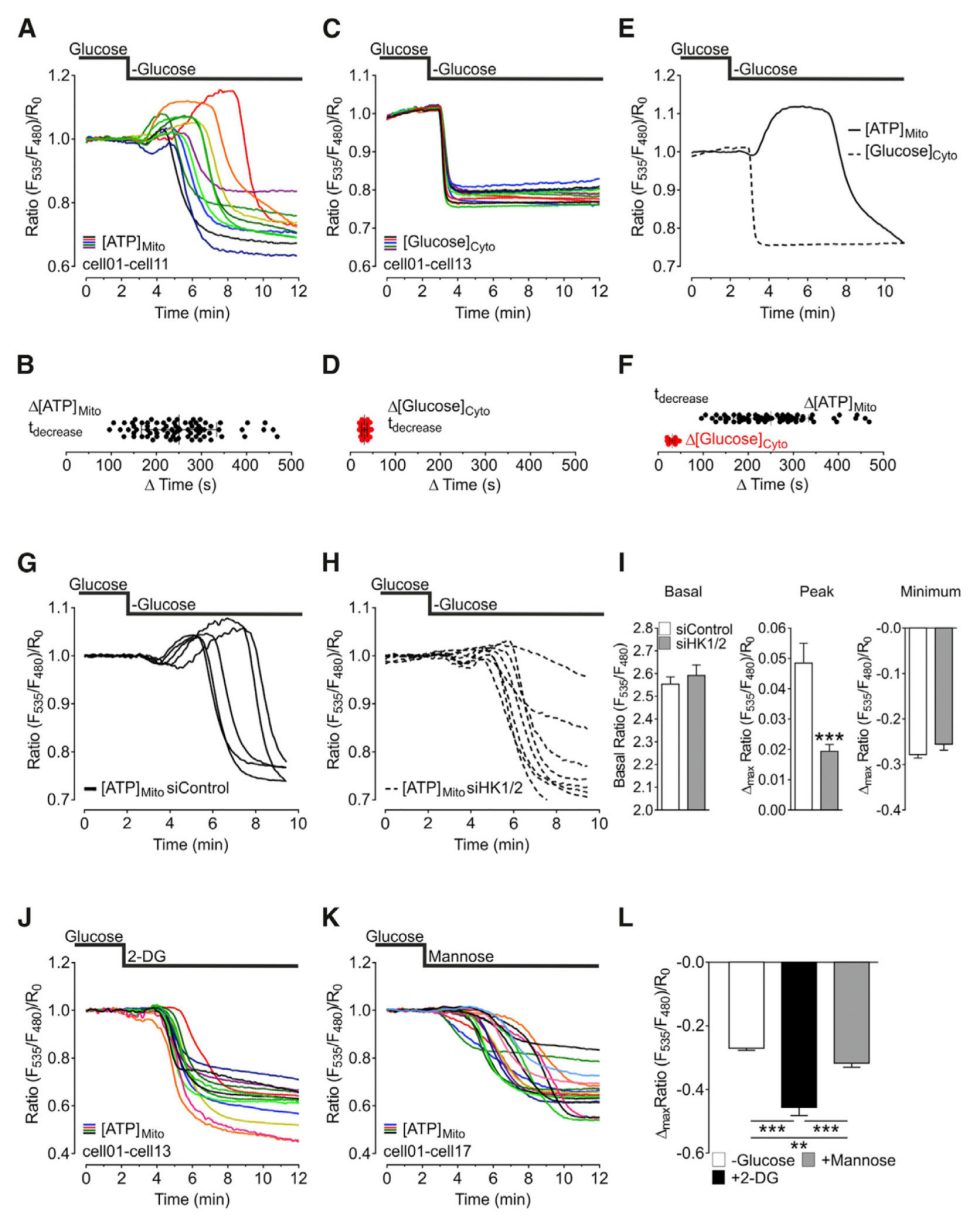
Transient Mitochondrial ATP Increase upon Glucose Deprivation Depends on Mitochondria-Located Hexokinase 1 and 2 Activities (A) Representative mitochondrial ATP responses to glucose depletion. See also Figure S1F. (B) Statistical analysis of the onset of mitochondrial ATP decrease (n = 6/76 cells; mean, SD). (C) Representative responses of cytosolic glucose levels to glucose withdrawal, measured with the glucose sensor FLII^12^Pglu-700μδ6. See also Figure S1G. (D) Statistical analysis of the onset of glucose depletion (n = 6/74 cells; mean, SD). (E) Comparison of representative time courses of cytosolic glucose (dashed line) and mitochondrial ATP (solid line). (F) Comparison of (B) and (D). (G) Representative mitochondrial ATP responses to glucose depletion in cells treated with control siRNA (siControl). (H) Representative mitochondrial ATP responses to glucose depletion in cells treated with siRNA against hexokinase 1 and 2 (siHK1/2). See also Figures S2 and S3. (I) Statistical analysis of mitochondrial ATP responses (left columns, basal levels; middle columns, peak height*;* right columns, minimum) to glucose depletion in cells treated with siRNA against hexokinase 1 and 2 (gray columns; n = 14/58 cells) compared to cells treated with control siRNA (white columns, n = 12/53 cells). Mean, SEM. ***p < 0.001 versus siControl. (J) Representative mitochondrial ATP responses to glucose substitution with 10 mM 2-DG. (K) Representative mitochondrial ATP responses to glucose substitution with 10 mM mannose. (L) Maximal mitochondrial ATP depletion after glucose removal only (white column; n = 24/124 cells) or substitution with 2-DG (black column; n = 7/31 cells) or mannose (gray column; n = 20/80 cells). Mean, SEM. **p < 0.01; ***p < 0.001.

The amplitude and kinetics of the transient ATP increase within HeLa cell mitochondria upon glucose removal showed a high cell-to-cell variability (Figures 2A, 2B, and 2F), probably pointing to different metabolic activities of individual cells. The signal differences within this cloned cell line were observed in cells on the same dish (Figure S1F) or among different dishes. In contrast, cytosolic glucose levels, which were measured with a genetically encoded glucose sensor (Takanaga et al., 2008), decreased instantly and uniformly upon glucose removal (Figures 2C, 2D, 2F, and S1G), and the glucose depletion preceded the mitochondrial ATP response (Figures 2E and 2F). We hypothesized that the mitochondrial peak ATP signal was linked to the activity of hexokinase (HK) enzymes for two reasons: first, the initial step of glucose metabolism is ATP-dependent phosphorylation of glucose by hexokinase enzymes. Second, hexokinases localize to the outer mitochondrial membrane (OMM) (Figure S2), where they interact with the OMM voltage-dependent anion channel (VDAC), suggesting that these enzymes consume mitochondria-derived ATP (Arora and Pedersen, 1988; Nelson and Kabir, 1986; Roberts and Miyamoto, 2015; Robey and Hay, 2006; Wilson, 2003). To directly investigate the role of the two predominant hexokinase isoforms hexokinase 1 (HK1) and hexokinase 2 (HK2) on the mitochondrial ATP increase upon glucose removal, the expression of both enzymes was partially silenced in HeLa cells using small interfering RNA (siRNA) (Figure S3A). While knockdown of HK1 and HK2 did not affect basal cytosolic ATP levels (Figure S3B), the amplitudes of the mitochondrial ATP peak upon acute glucose removal in cells treated with siRNAs against HK1 and HK2 were significantly reduced compared to respective controls (Figures 2G–2I). Next, we used super-resolution structural illumination microscopy (SIM) to investigate the subcellular localization of GFP tagged HK1 (HK1-GFP) and HK2 (HK2-GFP) in intact HeLa cells. For this purpose, cells expressing either HK1-GFP (Figure S2A) or HK2-GFP (Figure S2B) were co-transfected with mCherry-TOM22, a label of the OMM or co-stained with MitoTracker Red. These experiments showed that both HK1-GFP and HK2-GFP are exclusively located at the OMM of intact HeLa cells (Figure S2).

We then used another approach to investigate the relevance of hexokinase enzymes for the peak mitochondrial ATP signal. Glucose was substituted with either 2-DG or mannose, both of which remain phosphorylated by hexokinases. Indeed, glucose substitution with 2-DG (Figure 2J) or mannose (Figure 2K) did not produce mitochondrial ATP increases, but considerably reduced ATP levels within the organelles. Replacing glucose by 2-DG depleted mitochondrial ATP more efficiently and faster, compared to the removal of glucose or the exchange of glucose by mannose (Figures 2J–2L). While the addition of 2-DG induced rapid and quite uniform mitochondrial ATP depletions in all HeLa cells (Figure 2J), substitution of glucose with mannose provoked mitochondrial ATP drops with a broader range of kinetics (Figure 2K).

### Hexokinase Reaction May Be Reversible and Fuel Mitochondria with ATP in the Absence of Hexoses

**Figure 3:**
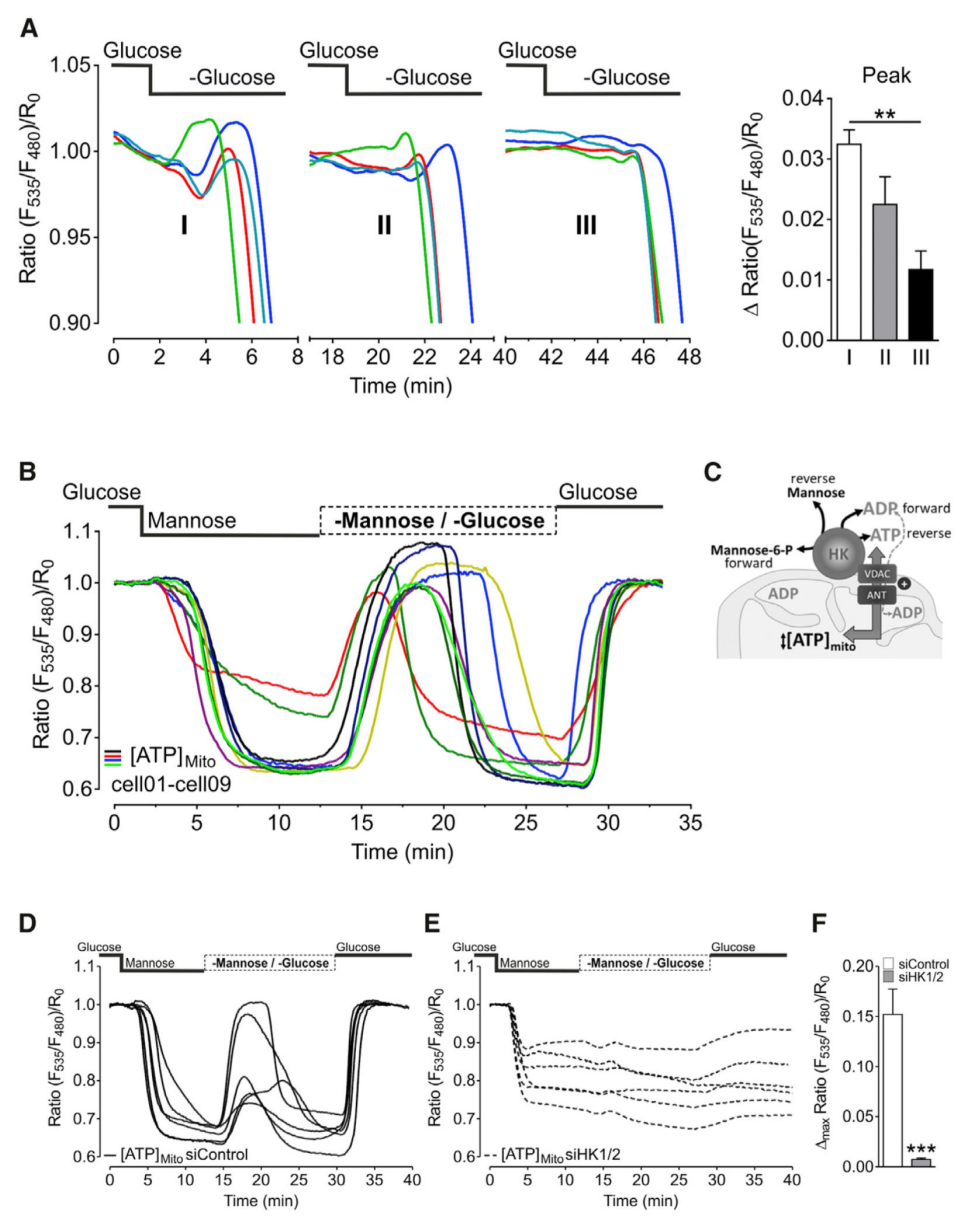
Hexokinase Reaction May Be Reversible and Fuel Mitochondria with ATP (A) Left panel: representative curves showing the transient rise of mitochondrial ATP and the onset of mitochondrial ATP depletion in response to repeated (three times in a row) glucose depletion in HeLa cells. Periods of glucose readdition (10 mM) are left out. Right panel: comparison of maximal FRET ratio increases of mtAT1.03 after first (white column, I), second (gray column, II), and third (black column, III) time glucose was removed (mean, SEM; n = 4/17 cells). **p < 0.01. (B) Representative mitochondrial ATP responses to glucose substitution with mannose and subsequent withdrawal of mannose (n = 5/25 cells). (C) Scheme showing reversed activity of mitochondria-located hexokinase (HK) upon removal of mannose. It is assumed that, in the presence of mannose, HK forms mannose 6-phosphate, which is converted back to mannose and ATP upon mannose removal. Thereby ATP is transported into the mitochondrial matrix via VDAC and a mitochondrial adenine nucleotide translocator (ANT). See also Figure S2. (D) Representative mitochondrial ATP responses to glucose substitution with mannose and subsequent withdrawal of mannose in cells treated with control siRNA (siControl). (E) Representative mitochondrial ATP responses to glucose substitution with mannose and subsequent withdrawal of mannose in cells treated with siRNAs against hexokinase 1 and 2 (siHK1/2). See also Figure S3. (F) Statistical analysis of mitochondrial ATP elevations in response to mannose withdrawal (cf. Figures 3D and 3E) in cells treated with siRNA against hexokinase 1 and 2 (gray columns; n = 10/32 cells) compared to cells treated with control siRNA (white columns; n = 10/22 cells). Mean, SEM. ***p < 0.001 versus siControl.

When we successively repeated the glucose depletion protocol in one cell, the mitochondrial ATP peak became less pronounced with each repetition (Figure 3A). Hence, we hypothesized that the mitochondrial peak ATP signal is the result of the reversal of the hexokinase reaction. Because of the lack of glucose, no substrate is available for the phosphorylation reaction. Therefore, hexokinases may transform glucose 6-phosphate to glucose, and thereby produce ATP, which is instantly transferred into mitochondria. The manifestation of the peak signal would depend on the glucose 6-phosphate reserves of a cell (Lucarelli et al., 2015). After repeated glucose depletion the mitochondrial ATP peak became less prominent (Figure 3A), indicating that glucose 6-phosphate pools remained empty during short phases of glucose readdition, although the glycolytic ATP production restored mitochondrial ATP levels (Figure 1B). Although we are not sure about this interpretation, we then applied another protocol to test this hypothesis. The ATP depletion protocol with glucose substitution by mannose (Figure 2K) must result in the accumulation of mannose 6-phosphate since mannose is phosphorylated but not further metabolized. Thus, we introduced a protocol, where we first induce the depletion of mitochondrial ATP and the accumulation of mannose 6-phosphate (like in Figure 2K) and then switch to sugar-free buffer. Now mannose 6-phosphate could be used as a substrate by hexokinases since no other hexokinase substrate was supplied in the buffer. Indeed, we observed a transient rise of mitochondrial ATP when we switched from mannose containing to sugar-free buffer (Figure 3B). Similar to the results from glucose withdrawal (Figure 2A), the mitochondrial ATP increases were rather heterogeneous from cell to cell. To further test whether this mitochondrial ATP elevation is accomplished by the reverse action of mitochondria-associated hexokinases (Figure 3C), we added and subsequently removed mannose from cells that were treated with siRNA against HK1 and HK2 (Figures 3D–3F). Under these conditions, mitochondrial ATP levels did not significantly increase upon mannose removal (Figures 3E and 3F). These experiments indicated that the mitochondria-located hexokinase enzymes can convert glucose 6-phosphate (or mannose 6-phosphate) to glucose (or mannose) and ATP. However, the cause for the transient ATP elevations remains uncertain. Aside from that, the HK knockdown experiments also revealed that, in cells reduced of HK1 and HK2, mitochondrial ATP did not reach the starting value after glucose readdition (Figure 3E). These results demonstrate that imaging mitochondrial ATP dynamics in real time can be used to detect a reduced glycolytic rate and capacity in cells depleted of HK1 and HK2.

### ATP Synthase in HeLa Cells Works in Reverse Mode and Does Not Contribute to Mitochondrial ATP Elevations upon Glucose or Mannose Removal

**Figure 4:**
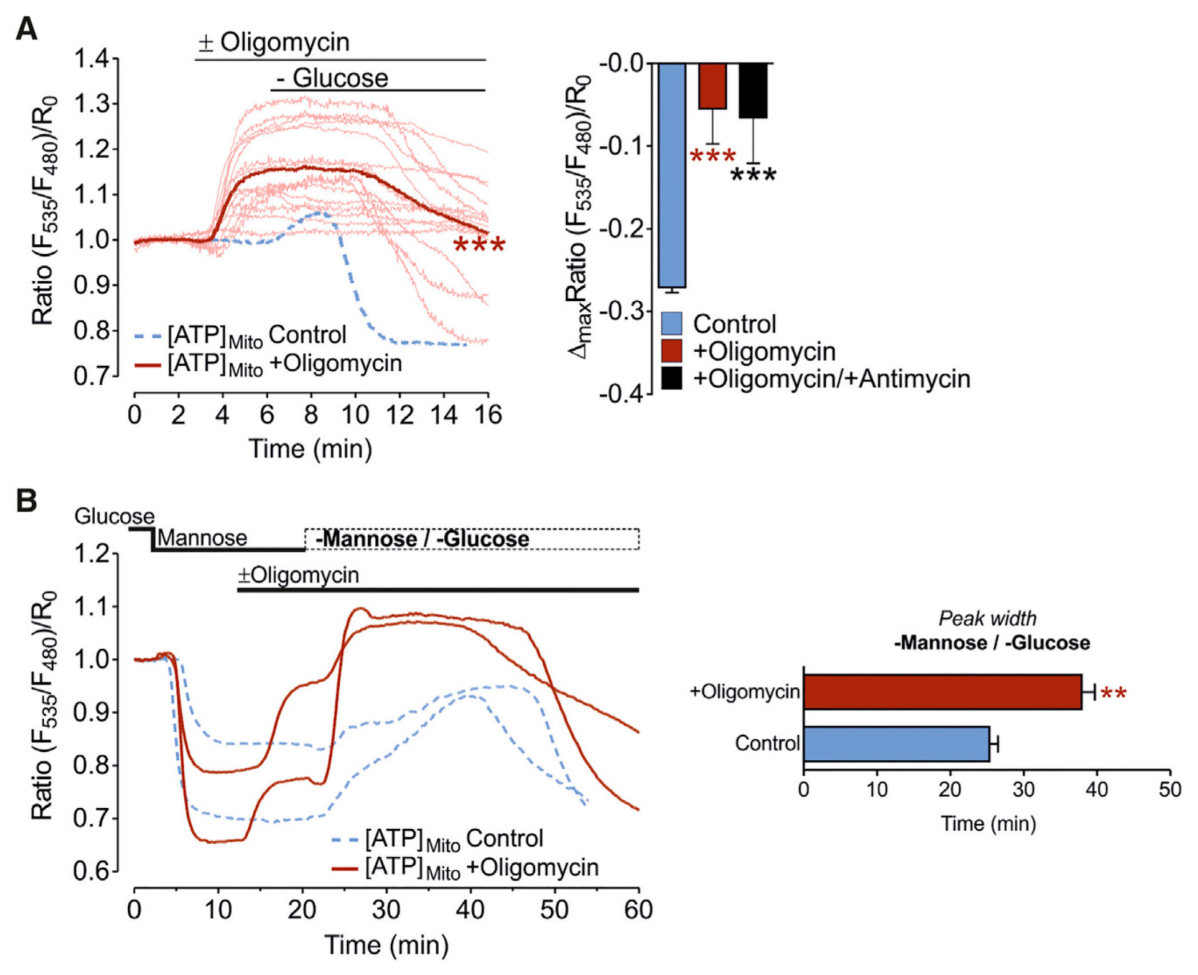
ATP Synthase in HeLa Cells Works in Reverse Mode and Does Not Contribute to Mitochondrial ATP Elevations upon Glucose or Mannose Removal (A) Left panel: mitochondrial ATP responses to oligomycin (2 μM) treatment, followed by glucose withdrawal in HeLa cells; single-cell responses (pale red lines) and mean curve (distinct red line). Blue-dashed line shows representative mitochondrial ATP response to glucose depletion in the absence of oligomycin. ***p < 0.001 versus control. Right panel: columns represent maximal FRET ratio changes of mtAT1.03 (mean, SEM) upon glucose depletion in the absence (blue column; n = 24/111 cells; control) and presence of oligomycin (2 μM; red column; n = 3/15 cells) or oligomycin (2 μM) and antimycin A (2.5 μM) (black column; n = 3/8 cells). ***p < 0.001 versus control. (B) Left panel: red solid curves show representative mitochondrial ATP responses to glucose substitution with mannose, followed by oligomycin (2 μM) addition and subsequent withdrawal of mannose in the presence of oligomycin; blue dashed curves show representative mitochondrial ATP responses to the same protocol, but without oligomycin (control). Right panel: statistical analysis of ATP elevations (peak width) after mannose withdrawal in the presence or absence (control) of oligomycin. **p < 0.01. See also Figure S4.

Next, we tested how inhibition of the ATP synthase affects mitochondrial ATP levels and the response to glucose withdrawal in HeLa cells. Inhibition of the ATP synthase with oligomycin elevated mitochondrial ATP levels and counteracted the organelle’s ATP depletion upon glucose removal (Figure 4A). This indicates that the ATP synthase is working in reverse mode extruding protons to maintain a negative membrane potential across the inner mitochondrial membrane. Measurements of the mitochondrial membrane potential using tetramethylrhodamine methyl ester (TMRM) and the intracellular pH with the pH probes SypHer or SypHer mt (Poburko et al., 2011) support this conclusion; oligomycin treatment caused a decrease of the pH in the mitochondrial matrix, caused an increase of the cytosolic pH, and reduced the mitochondrial membrane potential (Figures S4A–S4C). Accordingly, mitochondrial ATP depletion upon glucose removal also caused a drop in the mitochondrial membrane potential (Figure S4D).

We then investigated whether inhibition of the ATP synthase with oligomycin affects the ATP elevations caused by the switch from mannose to sugar-free buffer (Figure 3B). Oligomycin was added when mitochondrial ATP levels reached the minimal levels in mannose-containing buffer. Addition of oligomycin under these conditions induced an increase of mitochondrial ATP levels (Figure 4B, left panel). Nevertheless, the switch to sugar-free buffer still induced a further transient rise of mitochondrial ATP, which was lasting even longer than in the absence of oligomycin (Figure 4B). This experiment excludes that the mitochondrial ATP increase upon mannose removal is due to an activation of the ATP synthase.

### Mitochondrial ATP Depletion upon Glucose Deprivation Indirectly Correlates with Mitochondrial Respiration

**Figure 5:**
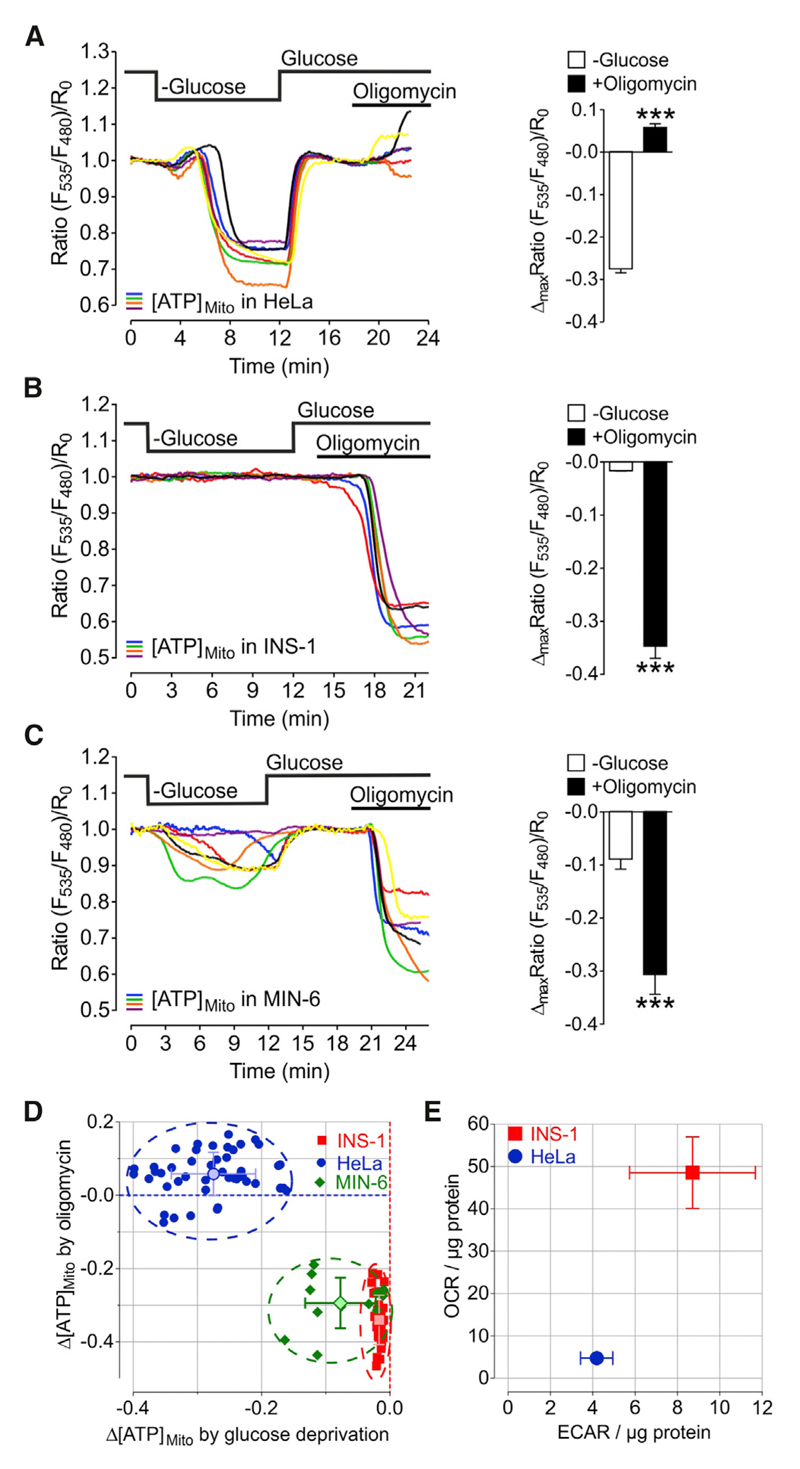
Mitochondrial ATP Depletion upon Glucose Deprivation Indirectly Correlates with Mitochondrial Respiration (A–C) Left panels: representative single-cell mitochondrial ATP responses of (A) HeLa, (B) INS-1, and (C) MIN-6 cells to glucose depletion and oligomycin (2 μM) treatment. Right panels: statistical analysis of maximal mitochondrial ATP depletion in response to glucose withdrawal compared to maximal changes after oligomycin treatment of (A) HeLa (n = 8/47 cells), (B) INS-1 (n = 4/34 cells), and (C) MIN-6 cells (n = 4/12 cells). Mean, SEM. ***p < 0.001. (D) Maximal mitochondrial ATP changes upon oligomycin (2 μM) treatment plotted against maximal mitochondrial ATP changes after glucose removal for HeLa (blue circles; n = 8/47 cells), INS-1 (red squares; n = 4/34 cells), and MIN-6 (green diamonds; n = 4/12 cells) cells. In addition, means ± SD are shown. (E) Oxygen consumption rate (OCR) plotted against extracellular acidification rate (ECAR) of HeLa and INS-1 cells measured by respirometry using Seahorse technology (mean ± SEM; n = 3 for both cell types). See also Figure S5.

Next, we investigated mitochondrial ATP dynamics in cells with high rates of oxidative phosphorylation, the rat and mouse pancreatic beta cell lines, INS-1 and MIN-6, respectively, compared to HeLa cells. We used a protocol, wherein we subsequently assessed glycolytic (glucose removal) and mitochondrial ATP production (oligomycin treatment) of individual cells during one measurement (Figures 5A–5D). Other than in HeLa cells (Figures 5A and 5D), glucose removal from INS-1 (Figures 5B and 5D) or MIN-6 cells (Figures 5C and 5D) had minimal effects on mitochondrial ATP levels. In contrast, treatment with oligomycin quickly and strongly reduced the mitochondrial ATP content of the pancreatic beta cells (Figures 5B–5D). As expected, compared to HeLa cells, INS-1 cells had higher oxygen consumption rates (OCRs) and extracellular acidification rates (ECARs), indicative of different metabolic activities of these cell types (Figures 5E and S5).

### Cellular Aging and Ablation of Mfn2 Specifically Alter Mitochondrial ATP Dynamics in MEFs

**Figure 6:**
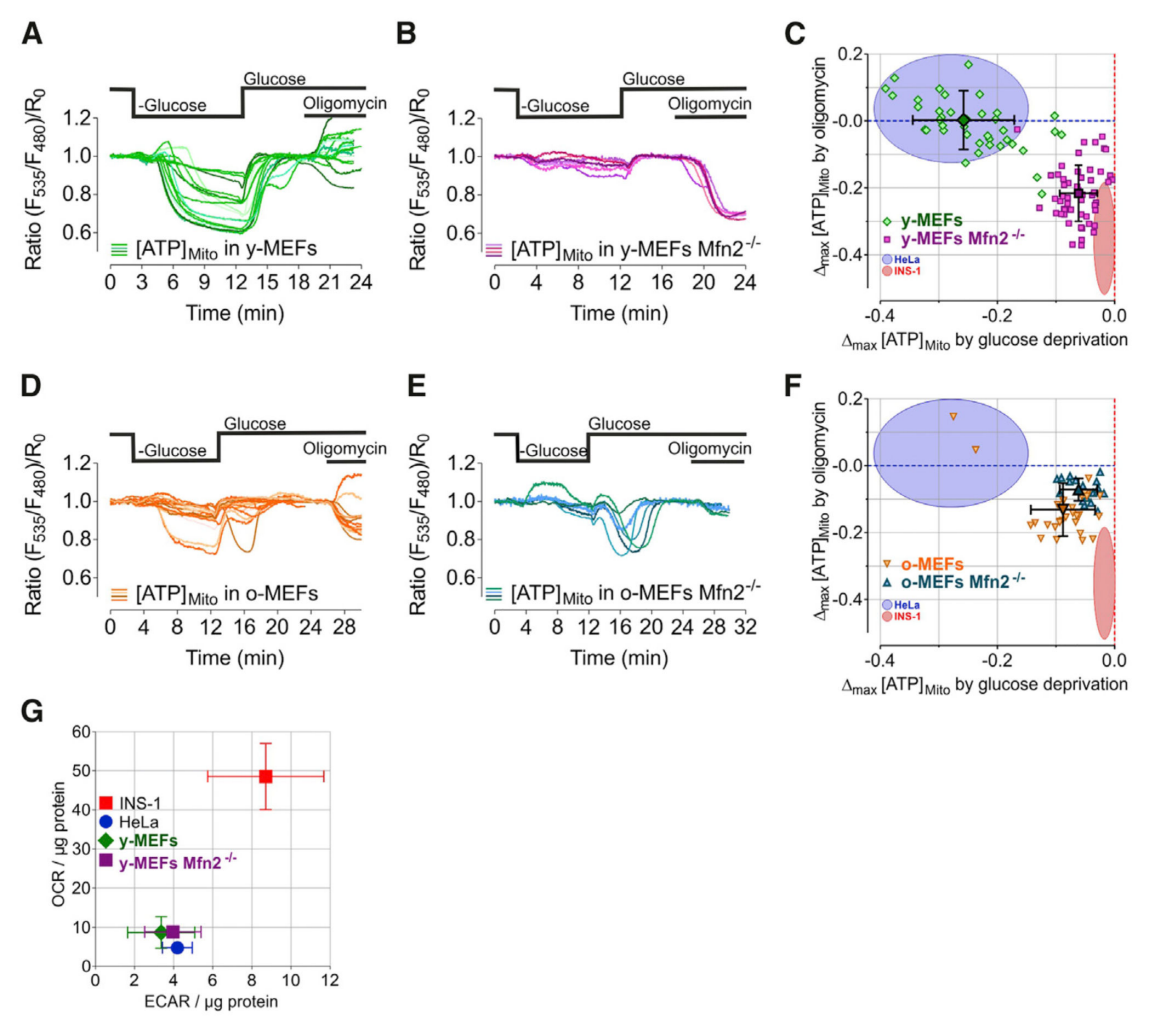
Cellular Aging and Ablation of Mfn2 Specifically Alter Mitochondrial ATP Dynamics in MEFs (A, B, D, and E) Representative mitochondrial ATP responses to glucose depletion and inhibition of the ATP synthase with oligomycin (2 μM) in (A) young/low passages MEFs (y-MEFs), (B) young Mfn2^–/–^ MEFs, (D) old/high passages MEFs (o-MEFs), and (E) old Mfn2^–/–^ MEFs. (C and F) Single-cell maximal mitochondrial ATP changes induced by oligomycin plotted against maximal ATP changes upon glucose depletion (mean, SD). (C) Comparison of young/low passages wild-type MEFs with young/low passages Mfn2^–/–^ MEFs. (F) Comparison of old/high passages wild-type MEFs with old/high passages Mfn2^–/–^ MEFs. (G) Oxygen consumption rates (OCR) plotted against extracellular acidification rates (ECARs) of young wild-type MEFs (y-MEFs) and young Mfn2^–/–^ MEFs compared to HeLa and INS-1 cells, determined by respirometry using Seahorse technology. Mean, SEM, n = 3. See also Figure S5.

Next, we used mouse embryonic fibroblasts (MEFs) to investigate whether or not aging had any impact on mitochondrial ATP dynamics as a sign of metabolic adaptations. Most young (low passages) MEFs from wild-type (WT) mice (y-MEFs) showed ATP alterations within mitochondria upon glucose removal quite similar to HeLa cells (Figures 6A and 6C). In contrast, mitochondrial ATP levels in aged (high passages; i.e., old) MEFs (o-MEFs) were less affected by glucose removal but clearly reduced by cell treatment with oligomycin, indicating enhanced oxidative phosphorylation in senescent MEFs (Figures 6D and 6F).

We then used the same approach to investigate the impact of mitofusin 2 (Mfn2) knockout on the metabolic activity of single cells. We chose Mfn2 because its role in mitochondria tethering to the ER and cellular metabolism is debated (de Brito and Scorrano, 2008; Filadi et al., 2015; Muñoz et al., 2013; Ngoh et al., 2012; Sebastián et al., 2012; Zorzano et al., 2015). MEFs from young Mfn2 knockout mice (y-MEFs Mfn2^–/–^) showed a rather moderate mitochondrial ATP depletion in response to acute glucose depletion, but ATP levels within mitochondria were strongly reduced by oligomycin (Figures 6B and 6C). In old Mfn2 knockout MEFs, no additional shift in metabolism could be seen compared to old wild-type MEFs (Figures 6E and 6F). These results indicate that Mfn2 ablation modifies the metabolic setting of cells in favor of oxidative phosphorylation. To depict the metabolic setting and activities of single cells, we again plotted the maximal mitochondrial ATP alterations in response to glucose starvation (x axis) against the maximal change after oligomycin treatment (y axis) (Figures 6C and 6F). This singlecell analysis showed distinct classes of metabolically dissimilar settings of young, aged, and Mfn2 knockout MEFs.

Notably, classical measurements of both the oxygen consumption rate (OCR) and extracellular acidification rate (ECAR) in populations of y-MEFs WT and y-MEFs Mfn2^–/–^ using respirometry (Seahorse) showed fewer differences in the metabolic activities (Figures 6G and S5) compared to the single-cell mitochondrial ATP imaging approach (Figures 6A–6F).

### Imaging of Mitochondrial ATP Dynamics in Single Cancer Cells Defines Distinct Metabolic Settings

**Figure 7:**
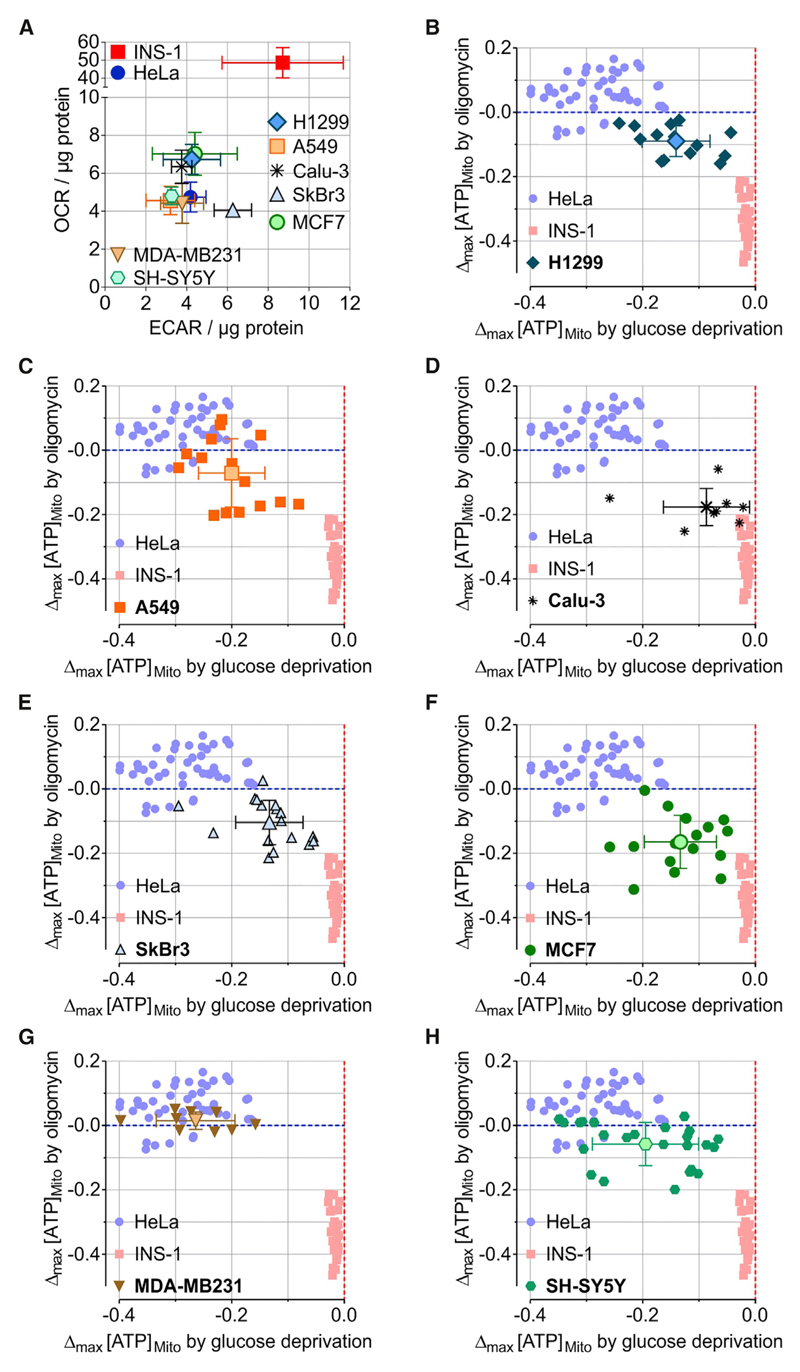
Imaging of Mitochondrial ATP Dynamics in Single Cancer Cells Defines Distinct Metabolic Settings (A) Oxygen consumption rate (OCR) and extracellular acidification rate (ECAR) of different cancer cell lines (H1299, A549, Calu-3, SkBr3, MCF7, MDA-MB231, and SH-SY5Y) and HeLa and INS-1 cells for comparison. Mean, SEM. n = 3. See also Figure S5. (B–H) Single-cell maximal mitochondrial ATP changes induced by oligomycin (2 μM) plotted against maximal ATP changes upon glucose depletion for (B) H1299, (C) A549, (D) Calu-3, (E) SkBr3, (F) MCF7, (G) MDA-MB231, and (H) SH-SY5Y cells, as well as HeLa and INS-1 cells for comparison (cf. Figure 5D). Means ± SD are shown. See also Figures S6 and S7.

The other major use of the mitochondrial ATP imaging approach we wanted to test was assessing the single-cell metabolic activities of different cancer cell types. Therefore, we cultured H1299, A549, and Calu-3 cells, which are frequently used human lung cancer cells. In addition, we used SkBr3, MCF-7, and MDAMB231 cells, which are well-characterized human breast cancer cell lines, as well as SH-SY5Y, a classic neuroblastoma cell model. All cancer cells had similar basal cytosolic glucose levels, which immediately decreased after glucose removal, pointing to high cellular glucose uptake and consumption (Figure S6). In line with other studies, these cancer cells of different origin showed rather low OCR (Figure 7A), the expected metabolic setting of cancer cells (low levels of oxidative phosphorylation, but enhanced glycolysis and lactate production). Then we imaged these cancer cells expressing the mitochondria-targeted ATP probe and quantified maximal FRET ratio changes in response to glucose removal and oligomycin. We again plotted respective data points against each other and compared them with those extracted from experiments with HeLa and INS-1 cells (Figures 7B–7H). These single-cell analyses showed that most cancer cells, such as H1299 (Figure 7B), A549 (Figure 7C), SkBr3 (Figure 7E), MCF-7 (Figure 7F), and SH-SY5Y (Figure 7H), showed large cell-to-cell variations in mitochondrial ATP dynamics (for single-cell traces, see Figure S7), indicating heterogeneous metabolic activities, a finding that remains undetectable using respirometry (Figures 7A and S5). In addition, these cancer cell types did not correspond with the metabolic setting of HeLa or INS-1 cells (Figures 7B, 7C, 7E, 7F, and 7H). However, most Calu-3 cells showed comparable mitochondrial ATP dynamics with INS-1 cells (Figure 7D), whereas only the breast cancer cell line MDA-MB231 displayed mitochondrial ATP dynamics similar to those of HeLa cells (Figure 7G). These imaging experiments point to distinct metabolic settings and activities in different human cancer cell types.

## Discussion

We visualized the metabolic activity of individual cells with the help of genetically encoded fluorescent ATP sensors, focusing on ATP dynamics within mitochondria of cancer cells. In the mitochondrial matrix, ATP concentrations change rapidly in response to experimental interventions such as glucose depletion. However, cytosolic and ER ATP levels are less dynamic. Although most cancer cell types do not produce much ATP via mitochondrial respiration (Zheng, 2012), our results indicate that mitochondria, as a distribution center for ATP, seem to be involved in the coordination of energy homeostasis in highly proliferating cancer cells. Blocking ATP synthase with oligomycin even elevated mitochondrial ATP levels in HeLa cells and counteracted ATP depletion upon glucose removal. While textbooks usually describe mitochondria as the “powerhouse of the cell,” because they are capable of generating most of the cellular ATP (Graier et al., 2007), rapidly proliferating cells are known to produce most ATP via the glycolytic degradation of glucose (Brand and Hermfisse, 1997). Beyond that, our experiments suggest that a lot of the ATP produced during aerobic glycolysis (i.e., the Warburg effect) is actually transferred into and consumed by mitochondria in cancer cells. Consequently, glucose withdrawal primarily affects the mitochondrial ATP pool, pointing to an import mechanism for ATP via either the adenine nucleotide translocase (ANT) or another ATP transporter, such as the APC (ATP-Mg/P_i_ carrier) (Maldonado et al., 2017). Following import, ATP is consumed to some extent by the F_1_F_O_ ATP synthase/ATPase. In various pathological conditions and diseases, such as Parkinson’s, Alzheimer’s, and motor neuron diseases, as well as stroke and heart attack, ATP consumption by mitochondria has been reported (Chinopoulos, 2011). It has been assumed that ATP depletion by the reverse action of the ANT and ATP synthase in respiration-deprived mitochondria represents a detrimental pathogenic mechanism, which can cause cell dysfunctions and death (Campanella et al., 2009; Jonckheere et al., 2012; Takeda et al., 2004). On the other hand, H^+^ pumping by the reverse ATP synthase was shown to counteract depolarization of impaired mitochondria (Campanella et al., 2008; McKenzie et al., 2007). But one rarely considers this reversal of activity as a cellular energetic advantage. Nevertheless, our data support the idea that steady import of ATP into mitochondria may be necessary to maintain a negative mitochondrial membrane potential because mitochondrial respiration is low in most cancer cells (Pelicano et al., 2006). Furthermore, the ATP train into mitochondria might prevent the repression of glycolysis by increasing ATP-to-ADP ratios in the cytosol and therefore help to keep the glycolytic rate high. ATP exchange between the cytosol and mitochondria might thus be crucial for the regulation of the cell’s metabolic activity. Our data further suggest that increased mitochondrial ATP uptake might even initialize enhanced cytosolic ATP production by glycolysis and the transition into the “Warburg” metabolism. In this context, the expression and regulation of different ANT isoforms and other ATP transporters probably plays an important role; high ANT2 expression levels, for example, are connected with a glycolytic metabolism and cancerogenesis (Chevrollier et al., 2011). Hence, we aim to assess the role of ATP transporters with regard to the findings presented in this work in future studies. Different from mitochondria, cytosolic ATP levels are kept rather stable, mainly by the regulatory activity of different energy stress sensors such as AMP-activated protein kinase (AMPK) (Herzig and Shaw, 2018). When the ATP-to-ADP or ATP-to-AMP ratio decreases, AMPK is activated and switches off energy-consuming processes, while activating energy-generating processes (Hardie and Lin, 2017).

Based on our observations with the widely used HeLa cancer cell line (Bonora et al., 2015), we aimed to define the metabolic profile for other cancer cell lines using mitochondrial ATP measurements in breast adenocarcinoma cells (MCF7, SkBr3, and MDA-MB231), lung adenocarcinoma cells (Calu-3, A549, and H1299) and neuroblastoma cells (SH-SY5Y). Compared to respirometry using the Seahorse technology, this approach allowed a better discrimination between the cell lines in terms of their preferred energy producing pathway. While OCR (as a measure for mitochondrial respiration) and ECAR (as a measure for glycolysis) were rather similar in all cancer cells lines, there were wider ranges of responses of mitochondrial ATP to glucose deprivation (measure for glycolytic ATP production) and oligomycin treatment (measure for mitochondrial ATP production). Thus, real-time measurements of mitochondrial ATP dynamics as a complementary tool to respirometry will certainly prove useful for bioenergetic analyses. Furthermore, the mitochondrial ATP curves may contain a lot more information about the mechanisms responsible for the metabolic setting of a cell. Experimental interventions and modified protocols, which may allow us to extract this information, are easily applicable in the case of ATP live cell imaging. An example is given by comparing the two breast cell cancer lines MCF7 and MDA-MB231. A high glycolytic rate is associated with increased tumor aggressiveness. Highly invasive MDA-MB231 cells are known to be more glycolytic than non-invasive MCF7 cells (Gatenby and Gillies, 2004; Pelicano et al., 2014). Our results using both respirometry and mitochondrial ATP measurements confirm these metabolic phenotypes. The mechanisms behind these observations and other questions related to cancer cell metabolism (e.g., related to the Warburg effect and aerobic glycolysis) may be addressed with the help of ATP live cell imaging.

We demonstrate that the measurement of mitochondrial ATP changes in response to glucose depletion and ATP synthase inhibition is a fast and reliable approach to characterize the metabolic state of cells. In general, the measurement of intracellular ATP and other metabolites using fluorescent sensors is a meaningful tool to investigate cellular processes by making them visible. A remarkable example is given in this work with the investigation of the hexokinase activity. Based on our data we speculate that hexokinase enzymes are important mediators between glycolytic ATP production and the mitochondrial ATP pool. Because of the localization of hexokinase 1 and 2 at the outer mitochondrial membrane and the interaction with the outer mitochondrial membrane voltage-dependent anion channel 1 (VDAC1), it has been suggested that they use mitochondrial ATP to phosphorylate their substrate (Arora and Pedersen, 1988; Nelson and Kabir, 1986; Roberts and Miyamoto, 2015; Robey and Hay, 2006; Wilson, 2003). Although some evidence favors this hypothesis, a clear proof is missing. More strikingly, our findings support the idea that hexokinases might reverse their action producing ATP in the absence of glucose. Despite the large negative standard free energy value of the hexokinase reaction (−16 kJ/mole), Chevrollier et al. (2011) proposed the reversal of this reaction in a review. However, experimental data showing ATP generation by mitochondria-located hexokinases in intact living cells have not been available so far. Our experiments indicate that the ATP produced by the hexokinase reaction is transported into the mitochondrial matrix. Thus, it is also likely that these enzymes indeed also obtain ATP from there to phosphorylate the substrate. Although the phosphorylation of glucose to glucose 6-phosphate is generally considered to be irreversible under physiological conditions (Meurer et al., 2016), the association of hexokinases with mitochondria and the coupling with the mitochondrial ATP pool may allow the reversal of the reaction, if intracellular glucose levels are strongly reduced, in particular if one considers that the enzymatic activity was measured *in vitro* with purified enzymes. Our experiments indeed suggest that a lack of substrate can reverse the hexokinase reaction, which momentarily fuels mitochondria with ATP by converting hexose 6-phosphate intermediates to hexoses. Hence, it is also tempting to speculate that such an unusual ATP generating pathway is important under pathological conditions to delay cell dysfunctions and death during phases of an insufficient substrate supply, for example, during transient vessel occlusions.

While our data strongly indicate that the transient increases of mitochondrial ATP are connected to mitochondria-associated hexokinases, the reversal of the hexokinase reaction is not the only possible explanation for our observations. It also seems plausible that the removal of any hexokinase substrate, such as glucose or mannose, prompts the enzyme to release ATP designated for the phosphorylation reaction into mitochondria. The magnitude of the mitochondrial ATP peak would then primarily depend on the activity of mitochondria-located hexokinases and mitochondrial ATP concentrations. Low mitochondrial ATP together with a depolarized mitochondrial membrane potential would favor ATP import above export (Chinopoulos, 2011), which would explain why stronger ATP increases were observed, when mitochondria were depleted of ATP. Anyhow, increased effort will be necessary to determine the connection between hexokinases and the mitochondrial ATP pool and the hexokinase mediated generation or release of ATP.

As discussed before, the role of different ATP transporters remains to be investigated also in this context. At first, the idea of a steady import of mitochondrial ATP into mitochondria does not go with the hexokinase enzymes taking mitochondrial ATP. However, several factors may allow such a scenario, such as the co-action of different ATP transporters, the organization of the inner mitochondrial membrane (e.g., cristae, inner boundary membrane), or the association with ATP producing or consuming processes (e.g., hexokinase reaction, ATP synthase activity). Moreover, the FRET ratio images representing mitochondrial ATP levels even suggest, that some areas within the mitochondrial matrix had higher or lower ATP concentrations, perhaps reflecting the existence of mitochondrial subcompartments with different ATP turnover or transport qualities. This is, however, very speculative and further experiments are necessary to investigate local ATP levels and dynamics within mitochondria and their subcompartments.

In a further set of experiments, we applied the same protocol which we used to characterize the different cancer cell lines to analyze the effects of Mfn2 knockout on energy metabolism. Mfn2 is involved in mitochondrial fusion (Chen et al., 2003). Its role in the formation of mitochondria-associated ER membranes (MAMs) is controversial. It could either serve as a tether (de Brito and Scorrano, 2008) or a distance keeper (Filadi et al., 2015) between the ER and mitochondria. Previous reports have disclosed the role of Mfn2 in cell metabolism (Zorzano et al., 2015). They show that the silencing of Mfn2 reduces oxygen consumption in different cell models and tissues. In contrast, it was found that the stable knockout of Mfn2 in MEFs leads to increased respiration (Kawalec et al., 2015). Kawalec et al. (2015) propose that this is due to an adaptation process of the knockout cells. It seems that the short-term effects of Mfn2 deprivation or silencing on cell metabolism are very different from long-term effects. However, unlike Kawalec et al. (2015), we were not able to clearly discriminate between Mfn2 wild-type and knockout cells using respirometry. This can be attributed to their use of an Oxygraph-2k (OROBOROS), which is more sensitive than the Seahorse technology when the OCR range is rather low. Moreover, whether cells are in suspension (OROBOROS) or adherent (Seahorse) may also influence cell metabolism.

Looking at subcellular ATP dynamics not only allows the fast and convincing determination of metabolic activities at the level of individual cells but also shows unexpected energy-converting pathways, warranting further research with multiple implications in health and disease.

## Star★Methods

### Key Resources Table

**Table 1:** 

REAGENT or RESOURCE	SOURCE	IDENTIFIER
Chemicals, Peptides, and Recombinant Proteins		
TransFast Transfection Reagent	Promega	Cat# E2431
2-Deoxy-D-glucose	Alfa Aesar	Cat# L07338; CAS 154-17-6
D-Mannose	Carl Roth	Cat# 4220; CAS 3458-28-4
Oligomycin A	Sigma Aldrich or Tocris	Sigma: Cat# 75351; CAS 579-13-5; Tocris: Cat# 4110; CAS 579-13-5
Antimycin A	Sigma Aldrich	Cat# A8674; CAS 1397-94-0
TMRM	Molecular Probes, Invitrogen	Cat# T668
MitoTracker Red-FM	Molecular Probes, Invitrogen	Cat# M22425
Critical Commercial Assays		
Seahorse XFe96 Extracellular Flux Assay Kit	Agilent	Cat# 102416-100
Total RNA Kit	Peqlab	Cat# 732-2868
High-Capacity cDNA Reverse Transcription Kit	Applied Biosystems	Cat# 4368814
QuantiFast SYBR Green RT-PCR kit	QIAGEN	Cat# 204154
Experimental Models: Cell Lines		
HeLa S3	ATCC	N/A
H1299	ATCC	N/A
SH-SY5Y	ATCC	N/A
INS-1 (832/13)	C.B. Newgard, Duke University School of Medicine, USA; Hohmeier et al., 2000	N/A
MIN-6	CellBank Graz	N/A
MDA-MB231	CellBank Graz	N/A
MCF7	CellBank Graz	N/A
SkBr3	CellBank Graz	N/A
Calu-3	CellBank Graz	N/A
A549	CellBank Graz	N/A
MEF (mouse embryonic fibroblasts)	Thomas Simmen, University of Alberta, Canada	N/A
MEF Mfn2^–/–^	Thomas Simmen, University of Alberta, Canada	N/A
Oligonucleotides		
HK1 forward primer 5′ -GACTCGCTTCAGGAAGGAGATG-3′	Invitrogen	N/A
HK1 reverse primer 5′ -ACATCTTGACTGTGGCTGTTGG-3′	Invitrogen	N/A
HK2 forward primer 5′ -GATTGTCCGTAACATTCTCATCGA-3′	Invitrogen	N/A
HK2 reverse primer 5′ -TGTCTTGAGCCGCTCTGAGAT-3′	Invitrogen	N/A
Hexokinase 1 siRNA	Santa Cruz Biotechnology	Cat# sc-39044
Hexokinase 2 siRNA	Santa Cruz Biotechnology	Cat# sc-35621
Recombinant DNA		
pcDNA3.1 AT1.03	H. Imamura; Imamura et al., 2009	N/A
pcDNA3.1 mtAT1.03	H. Imamura; Imamura et al., 2009	N/A
pcDNA3.1 ERAT1.03	our lab; Vishnu et al., 2014	N/A
pcDNA3.1 FLII^12^Pglu-700μδ6	W. Frommer; Takanaga et al., 2008	Addgene # 17866
pSypHer-cyto	N. Demaurex; Poburko et al., 2011	Addgene # 48250
pSypHer-mito	N. Demaurex; Poburko et al., 2011	Addgene # 48251
FLHKI-pGFPN3	H. Ardehali; Sun et al., 2008	Addgene # 21917
FLHKII-pGFPN3	H. Ardehali; Sun et al., 2008	Addgene # 21920
Software and Algorithms		
Live Acquisition 2	TILL Photonics	N/A
Offline Analysis	TILL Photonics	N/A
MetaMorph	Molecular Devices	RRID: SCR_002368
Microsoft Excel	Microsoft	RRID: SCR_016137
GraphPad Prism5	GraphPad	RRID: SCR_002798
Nikon Nis-Elements	Nikon	RRID: SCR_014329
Fiji/ImageJ	Schneider et al., 2012	https://fiji.sc/
SIM image analysis macro	B. Gottschalk, this work e-mail: benjamin.gottschalk@medunigraz.at	N/A

### Contact for Reagent and Resource Sharing

Further information and requests for resources and reagents should be directed to and will be fulfilled by the Lead Contact, Roland Malli (roland.malli@medunigraz.at).

### Experimental Model and Subject Details

HeLa S3, H1299, and SH-SY5Y cells come from ATCC; MIN-6, MDA-MB231, MCF7, SkBr3, Calu-3 and A549 cells from the Core Facility Alternative Biomodels and Preclinical Imaging, Medical University of Graz (Graz, Austria). INS-1 cells were obtained from C.B. Newgard, Department of Pharmacology and Cancer Biology, Duke University School of Medicine, USA (Hohmeier et al., 2000). Mouse Embryonic Fibroblasts (MEFs) were a gift from Thomas Simmen, Department of Cell Biology, University of Alberta, Canada.

HeLa, MCF7, MDA-MB231, SkBr3, H1299 and MEF cells were grown in Dulbecco’s modified Eagle’s medium (DMEM) containing 10% FCS, 100 U/mL penicillin, 100 μg/mL streptomycin, 2.5 μg/mL amphotericin B, and 2 mM glutamine. A549, Calu-3, and SH-SY5Y cells were cultivated in a 1:1 mixture of Ham’s F12 medium and DMEM supplemented with 10% FCS, 100 U/mL penicillin, 100 μg/mL streptomycin, 2.5 μg/mL amphotericin B and 2 mM glutamine. INS-1 cells were grown in GIBCO RPMI medium 1640 supplemented with 10% FCS. MIN-6 cells were cultured in DMEM supplemented with 25 mM D-glucose, 10 mM HEPES, 10% FCS, 1 mM sodium pyruvate, 50 μM b-mercaptoethanol, 100 U/mL penicillin and 100 μg/mL streptomycin. Cell culture substances were obtained from Life Technologies (Vienna, Austria) and Carl Roth (Karlsruhe, Germany). All cells were grown at 37°C with 5% CO_2_.

### Method Details

#### Transfection

For the experiments, cells were seeded in 6-well plates with (for microscopy) or without (RNA isolation) 30 mm imaging dishes. They were transiently transfected at a confluence of 60 to 70% one to two days before the measurement. The transfection mix contained (per well): 1 mL DMEM (without serum and antibiotics), 2.5 μL TransFast transfection reagent (Promega, Madison, WI, USA) and 1.5 μg plasmid DNA encoding the respective fluorescent sensor and/or 100 nM siRNA. The transfection mix was replaced with full culture medium 6 to 12 hours after transfection. The siRNAs against hexokinase 1 and hexokinase 2 were obtained from Santa Cruz Biotechnology (Heidelberg, Germany). The siRNAs are pools of three target-specific 19 nucleotide siRNAs. Control siRNA was obtained from Microsynth (Balgach, Switzerland).

ATP sensors were a gift from Hiromi Imamura, Kyoto University, Kyodai Graduate School of Biostudies, Japan (Imamura et al., 2009). FLHKI-pGFPN3 and FLHKII-pGFPN3 were a gift from Hossein Ardehali (Addgene plasmid # 21917 and # 21920) (Sun et al., 2008). pcDNA3.1 FLII^12^Pglu-700μδ6 was a gift from Wolf Frommer (Addgene plasmid # 17866) (Takanaga et al., 2008). SypHer and SypHer mt were a gift from Nicolas Demaurex (Addgene plasmid # 48250 and # 48251).

#### Microscopy

For all imaging experiments, cells were equilibrated in loading buffer for one hour. Loading buffer contained: 2 mM CaCl_2_, 138 mM NaCl, 5 mM KCl, 1 mM MgCl_2_, 10 mM D-glucose, 2 mM L-glutamine, 10 mM HEPES, 2.6 mM NaHCO_3_, 0.44 mM KH_2_PO_4_, 0.34 mM Na_2_HPO_4_, 0.1% vitamins, 0.2% essential amino acids, 1% penicillin-streptomycin, pH adjusted to 7.4 with NaOH. All experiments were performed at room temperature in ambient atmosphere. For the life cell imaging experiments, cells were placed in a flow chamber. A gravity-based perfusion system (NGFI, Graz, Austria) in combination with a vacuum pump (Chemistry diaphragm pump ME 1c, Vacuubrand, Wertheim, Germany) allowed the steady perfusion of the cells with fresh buffer and the switching between different buffers. Standard physiological buffer contained: 2 mM CaCl_2_, 138 mM NaCl, 5 mM KCl, 1 mM MgCl_2_, 10 mM D-glucose, 10 mM HEPES, pH adjusted to 7.4 with NaOH. For glucose-free conditions, 10 mM D-mannitol (Sigma Aldrich, Vienna, Austria) was added instead of glucose. If required, glucose was replaced by 2-deoxy-D-glucose (2-DG, Alfa Aesar, ThermoFisher, Karlsruhe, Germany) or D-mannose (Carl Roth, Karlsruhe, Germany). Oligomycin and antimycin A were dissolved from 10 mM stock solutions (in DMSO). Oligomycin was obtained from Tocris (Bristol, UK) or Sigma Aldrich (Vienna, Austria), Antimycin A was from Sigma Aldrich (Vienna, Austria). Other chemicals were from Carl Roth (Karlsruhe, Germany).

Cells were selected randomly for the measurement, the total numbers of cells measured for each experiment are indicated in the figure legends. The measurement was performed with an iMic inverted and advanced fluorescent microscope using a x40 magnification objective (alpha Plan Fluor x40, Zeiss, Göttingen, Germany) with a motorized sample stage (TILL Photonics, Graefling, Germany). For control and acquisition, the software Live Acquisition 2 (TILL Photonics) was used. CFP/YFP FRET sensors were excited at a wavelength of 430 nm; emission was collected simultaneously at 535 and 480 nm using an optical beam-splitter (Dichroic 69008ET-ECFP/EYFP/mCherry). Data processing was performed with the Offline Analysis application (TILL Photonics). The mitochondrial membrane potential was measured with the fluorescent dye TMRM (Molecular Probes, Invitrogen, Eugene, OR, USA). TMRM was visualized at an excitation of 550 nm and emission of 575 nm.

The SIM-setup used is composed of a 405 nm, 488 nm, 515 nm, 532 nm and a 561 nm excitation laser introduced at the back focal plane inside the SIM-box with a multimodal optical fiber. For super-resolution, a CFI SR Apochromat TIRF 100x-oil (NA 1.49) objective was mounted on a Nikon-Structured Illumination Microscopy (N-SIM^®^) System with standard wide field and SIM filter sets and equipped with two Andor iXon3^®^ EMCCD camera mounted to a Two Camera Imaging Adaptor (Nikon Austria, Vienna, Austria). Cells were incubated for 40 min with Mitotracker Red-FM in loading buffer prior to imaging and washed twice with loading buffer. GFP was excited at 488 nm, MitoTracker Red was excited at 561 nm. For calibration and reconstruction of SIM images, the Nikon software Nis-Elements was used. To align both channels for parallel dual color experiments NIS-Elements Two-CAM registration was used taking the TetraSpeck bead samples. Image analysis was done using a custom-made ImageJ macro. Cells were selected by hand within the SIM images. Images were background corrected with an ImageJ Plugin (Mosaic Suite, background subtractor, NIH). The colocalization coefficients Pearson and Manders 1 and 2 were determined on a single cell basis with the ImageJ (Schneider et al., 2012) coloc 2 tool. Channel 1 represents the GFP and channel 2 the mCherry label. While the Pearson coefficient was determined with not thresholded data, the Manders 1 and 2 coefficients were determined on the basis of Costes thresholded images.

#### mRNA isolation and qRT-PCR

For qRT-PCR total RNA was isolated with a total RNA isolation kit (Peqlab, Erlangen, Germany). For reverse transcription, a cDNA synthesis kit (Applied Biosystems, Foster City, CA, USA) was used. For qRT-PCR, the QuantiFast SYBR Green RT-PCR kit (QIAGEN, Hilden, Germany) was used. Relative gene expression was normalized to human GAPDH (QuantiTect; QIAGEN). The reaction was performed on a LightCycler 480 (Roche Diagnostics, Vienna, Austria). Primers were obtained from Invitrogen (Vienna, Austria); their sequences were as follows (5′-3′): HK1 forward primer GACTCGCTTCAGGAAGGAGATG, HK1 reverse primer ACATCTTGAC TGTGGCTGTTGG, HK2 forward primer GATTGTCCGTAACATTCTCATCGA, HK2 reverse primer TGTCTTGAGCCGCTCTGAGAT.

#### Measurement of mitochondrial respiration

One day before the experiment cells were plated on XF96 polystyrene cell culture microplates (Seahorse^®^, Agilent, CA, USA). They had to be 100% confluent on the day of the experiment. Before the measurement cells were washed and incubated in XF assay medium supplemented with 1 mM sodium pyruvate, 2 mM glutamine and 5.5 mM D-glucose. An XF96 extracellular flux analyzer was used to measure oxygen consumption rate (OCR) and extracellular acidification rate (ECAR). OCR (pmol O_2_/min) and ECAR (mpH/min) values were normalized to protein content.

### Quantification and Statistical Analysis

For data analysis, Microsoft Excel (Redmond, WA, USA) and GraphPad Prism5 (GraphPad Software Inc.) were used. In case of both the FRET acceptor YFP and FRET donor CFP intensities, the respective background signals were subtracted. Then the YFP/CFP ratio was calculated. In case of live cell imaging data, curve fitting was used to correct for bleaching. Representative ratio images shown in Figure 1A and Video S1 were created using MetaMorph microscopy automation and image analysis software (Molecular Devices, Sunnyvale, CA, USA). Statistical analysis was performed with GraphPad Prism5 using either unpaired Student’s t test or one-way ANOVA with Tukey’s Multiple Comparison Test. n represents the number of independent experiments (at least three), and is indicated in the figure legends; the total number of measured cells is also indicated (e.g., n = 5/42 cells means 5 experiments with a total of 42 cells).

## Supplementary Material

Supplemental Information includes seven figures and one video and can be found with this article online at https://doi.org/10.1016/j.celrep.2018.09.027.

Figures S1-S7

## Highlights

- Subcellular ATP imaging reveals the metabolic setting of individual cells
- In cancer cells, mitochondrial ATP levels respond to glucose withdrawal
- Mitochondria-associated hexokinases link glycolysis to the mitochondrial ATP pool
- Mitochondrial ATP measurements are a useful tool for metabolic analyses
