# Supplementary material for: Real-Time Imaging of Mitochondrial ATP Dynamics Reveals the Metabolic Setting of Single Cells
Source: Cell Rep. Author manuscript; Available in PMC 2019 Apr 9. (PMC6456002; doi:10.1016/j.celrep.2018.09.027)
Supplement: Figures S1-S7 [file NIHMS82547-supplement-Figures_S1-S7.pdf]

Cell Reports, Volume *25*

Supplemental Information

Real-Time Imaging of Mitochondrial ATP Dynamics Reveals the Metabolic Setting of Single Cells

Maria R. Depaoli, Felix Karsten, Corina T. Madreiter-Sokolowski, Christiane Klec, Benjamin Gottschalk, Helmut Bischof, Emrah Eroglu, Markus Waldeck-Weiermair, Thomas Simmen, Wolfgang F. Graier, and Roland Malli

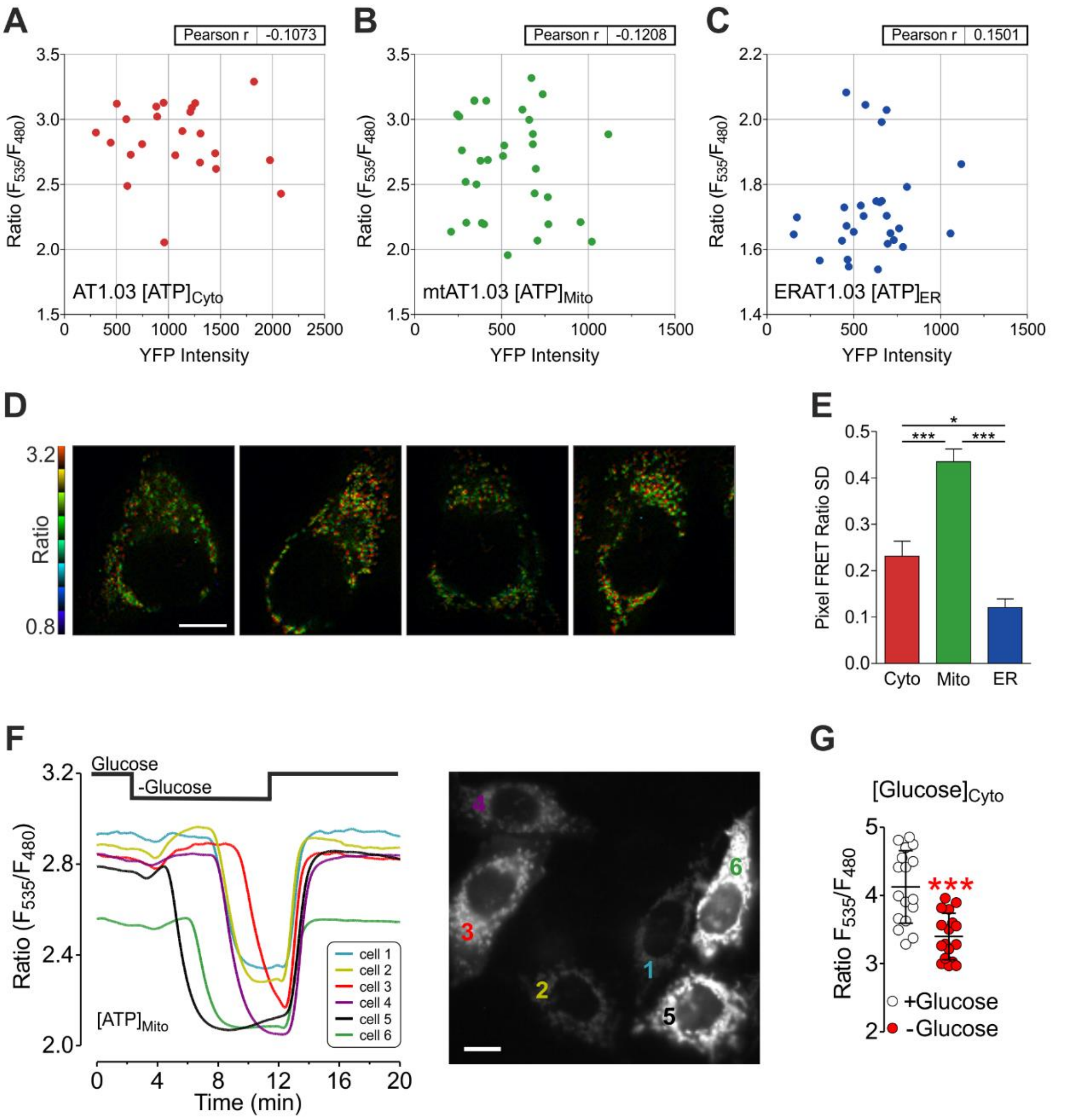

Figure S1. Details on intracellular ATP and glucose levels. Related to Figure 1 and 2. (A-C) Basal FRET ratios of **(A)** cytosolic ATP sensor AT1.03, **(B)** mitochondrial ATP sensor mtAT1.03, and **(C)** ER ATP sensor ERAT1.03, plotted against YFP fluorescence intensity. The Pearson correlation coefficient is indicated. **(D)** Representative FRET ratio images of mitochondrial ATP sensor mtAT1.03 under basal conditions. Scale bar represents 20 µM. **(E)** Standard deviation (SD) (mean, SEM) of pixel intensities of FRET ratio images of cytosolic (n=4 cells), mitochondrial (n=6 cells) and ER-targeted (n=6 cells) ATP probes (c.f. Figure 1). * $p < 0.05$; *** $p < 0.001$ **(F)** Responses of mitochondrial ATP levels to glucose depletion of single cells on one dish. Cell 1 to 6 are labeled on the corresponding microscopic image. Scale bar represents 20 µM. **(G)** FRET ratio levels of cytosolic glucose sensor FLII^{12}Pglu-700µδ6, corresponding to glucose levels, in HeLa cells, in the presence of glucose (10 mM) and after glucose depletion. Mean, SD, n=3/17 cells. *** $p < 0.001$.

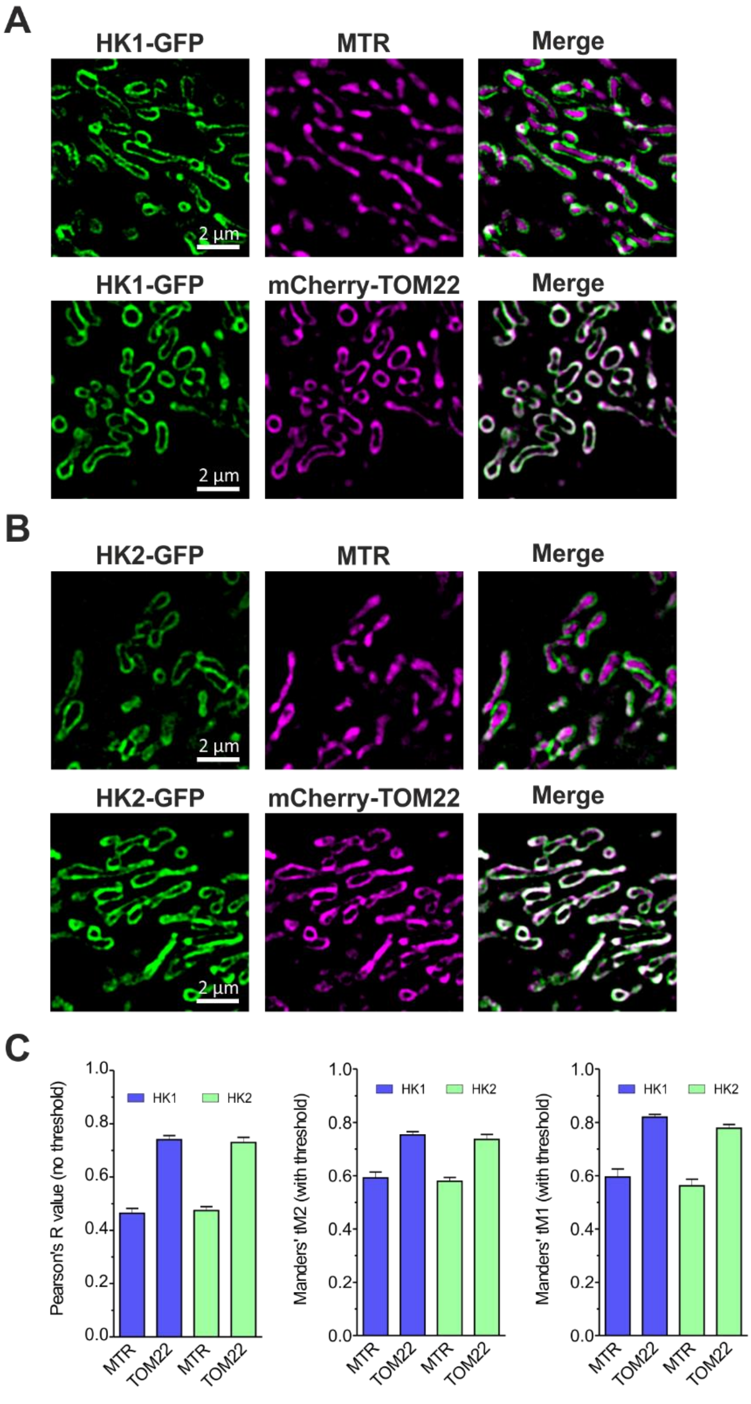

Figure S2. Hexokinase (HK) 1 and 2 localization in HeLa cells. Related to Figure 2 and 3. (A,B) Representative pictures of hexokinase co-localization experiments with MitoTracker Red FM (MTR) and the OMM marker mCherry-Tom22. **(A)** HK1-GFP and **(B)** HK2-GFP. **(C)** Quantitative analysis of colocalization coefficients Pearson and Manders 1 and 2. Channel 1 represents the GFP and channel 2 the mCherry label.

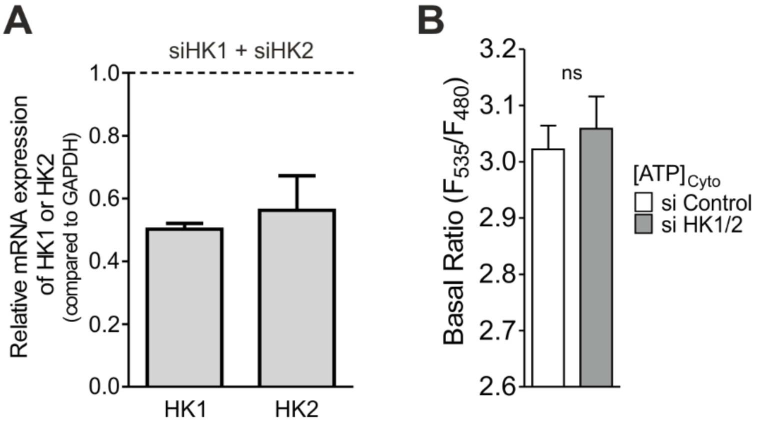

Figure S3. Hexokinase (HK) 1 and 2 knock-down in HeLa cells. Related to Figure 2 and 3. (A) Quantification of knock-down efficiency of HK1 or HK2 in HeLa cells after treatment with specific siRNAs via real-time PCR using GAPDH as a reference gene. Mean, SEM, n=3. **(B)** Basal FRET ratio of cytosolic ATP probe AT1.03 in cells treated with siRNA against hexokinase 1 and 2 (gray column, n=4/22 cells), compared to cells treated with control siRNA (white column, n=4/16 cells). Mean, SEM, ns not significant.

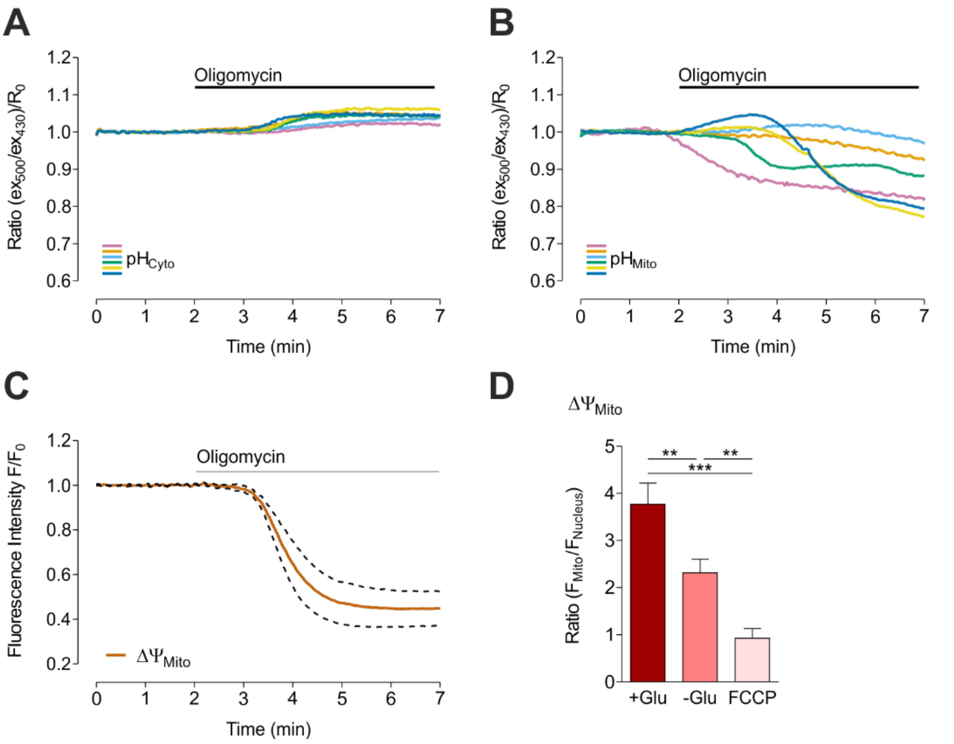

Figure S4. Effects of mitochondrial ATP synthase inhibition on intracellular pH and mitochondrial membrane potential. Related to Figure 4. (A) Representative FRET ratio signals of cytosolic pH probe SypHer in response to oligomycin induced inhibition of the ATP synthase. **(B)** Representative FRET ratio time courses of mitochondria targeted SypHer in response to oligomycin treatment. **(C)** Effect of oligomycin treatment on TMRM fluorescence intensity as a measure for the mitochondrial membrane potential ($\Delta\Psi_{Mito}$) (mean curve ± SEM). **(D)** Effect of glucose removal on the mitochondrial membrane potential ($\Delta\Psi_{Mito}$). Ratio between TMRM fluorescence intensities in mitochondria and nucleus in the presence of glucose (mean, SEM), after 10 minutes in glucose free buffer and after FCCP (1 µM) treatment. ** $p < 0.01$; *** $p < 0.001$.

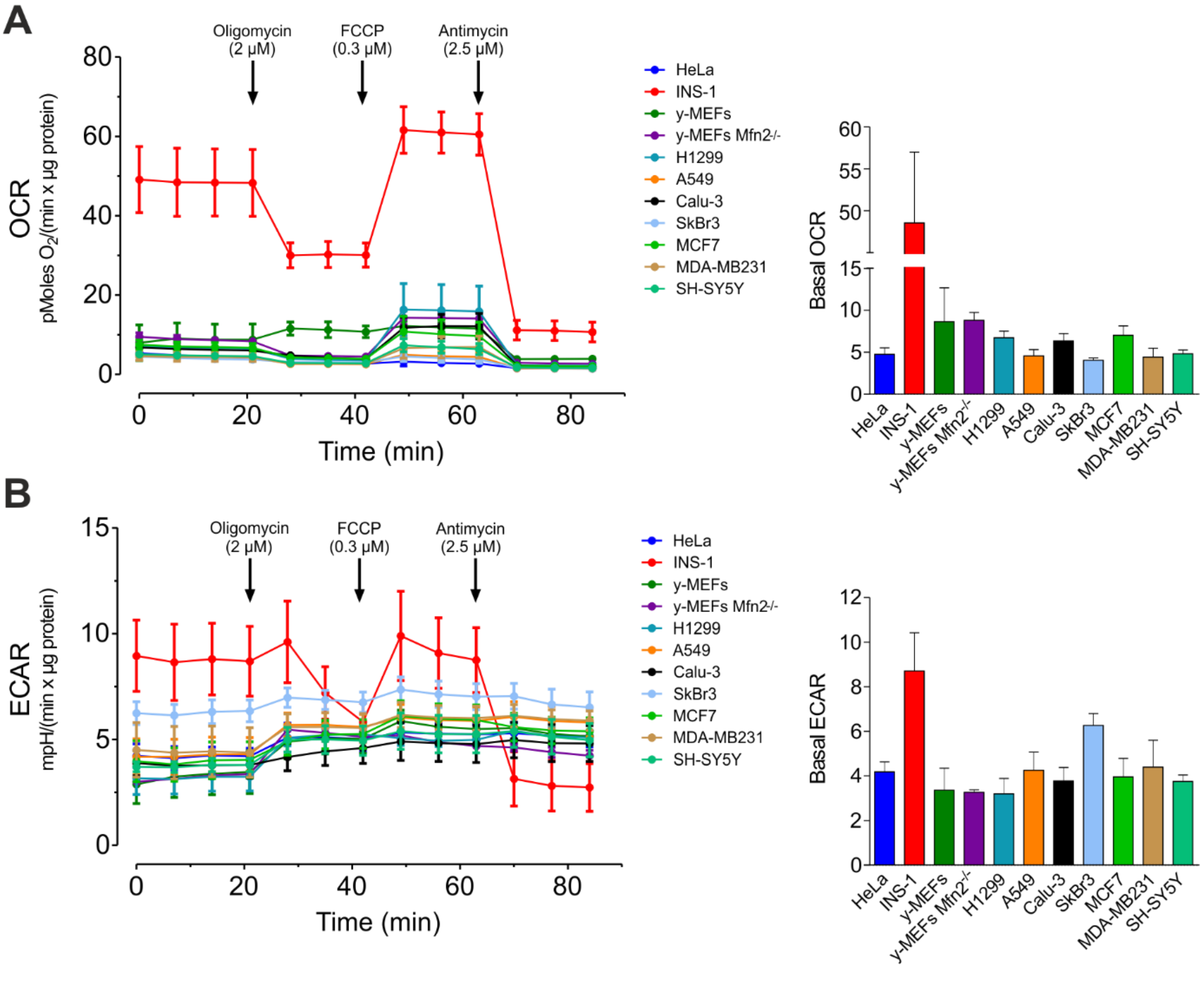

Figure S5. Oxygen consumption rates (OCR) and extracellular acidification rates (ECAR) of different cell lines. Related to Figures 5, 6 and 7. (A) OCR and **(B)** ECAR of HeLa and INS-1 cells, wild-type and Mfn2$^{-/-}$ MEFs, lung cancer cells (H1299, A549, Calu-3), breast cancer cells (SkBr3, MCF7, MDA-MB231) and neuroblastoma cells (SH-SY5Y). Oligomycin (2 μM), antimycin A (2.5 μM) and FCCP (0.3 μM) were injected as indicated. Bar graph shows basal OCR and ECAR values, respectively.

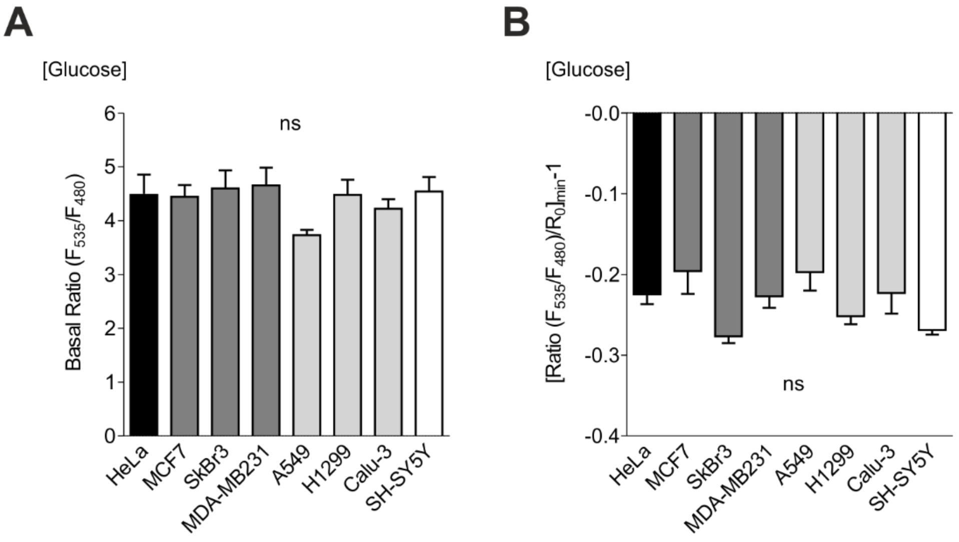

Figure S6. Different cancer cell lines have similar basal glucose levels, which decrease upon glucose removal. Related to Figure 7. (A) Bars represent basal FRET ratio signals of the cytosolic glucose sensor FLII^{12}Pglu-700μδ6 in different cancer cell lines (as indicated) in the presence of 10 mM glucose in the extracellular medium (mean, SEM). ns not significant. **(B)** Maximal FRET change of FLII^{12}Pglu-700μδ6 glucose sensor upon complete removal of glucose in different cancer cell lines (as indicated) (mean, SEM). ns not significant.

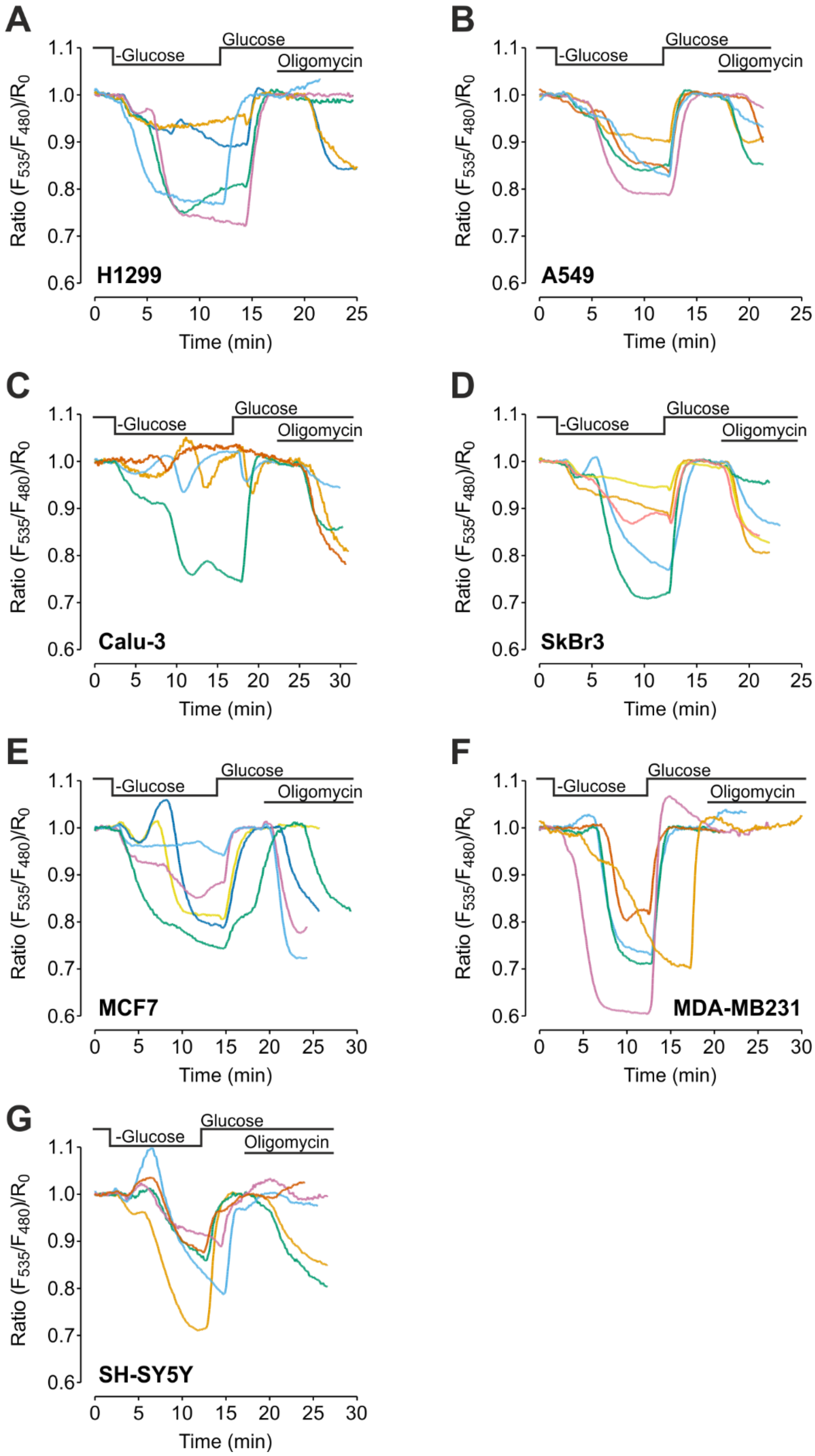

Figure S7. Single cell responses of mitochondrial ATP levels to glucose deprivation in different cancer cell lines. Related to Figure 7. Representative mitochondrial ATP curves of **(A)** H1299, **(B)** A549, **(C)** Calu-3, **(D)** SkBr3, **(E)** MCF7, **(F)** MDA-MB231, **(G)** SH-SY5Y cancer cells.
